# The effectiveness of postoperative rehabilitation interventions that include breathing exercises to prevent pulmonary atelectasis in lung cancer resection patients: a systematic review and meta-analysis

**DOI:** 10.1186/s12890-023-02563-9

**Published:** 2023-07-27

**Authors:** Jun Wang, Na Deng, Fang Qi, Qingbo Li, Xuegang Jin, Huiling Hu

**Affiliations:** 1 Department of Rehabilitation Medicine, The Second Xiangya Hospital of Central South University, Renmin Road No. 139, Furong District, Changsha, 410000 Hunan China; 2 Department of Adult Rehabilitation, Xiangya Boai Rehabilitation Hospital, Changsha, Hunan China; 3 Hunan University of Traditional Chinese Medicine, Changsha, Hunan China; 4 Hunan Children’s Hospital, Rehabilitation Center, Changsha, Hunan China; 5 Qinhuangdao Hospital of Traditional Chinese Medicine, Qinhuangdao, Hebei China

**Keywords:** Lung cancer, Postoperative rehabilitation intervention, breathing exercises, Postoperative pulmonary complication, Lung function

## Abstract

**Background:**

The main aim of this systematic review was to determine the effectiveness of postoperative rehabilitation interventions that include breathing exercises as a component to prevent atelectasis in lung cancer resection patients.

**Methods:**

In this review, we systematically and comprehensively searched the Cochrane Library, PubMed, EMBASE, and Web of Science in English and CNKI and Wanfang in Chinese from 2012 to 2022. The review included any randomized controlled trials focusing on the effectiveness of postoperative rehabilitation interventions that include breathing exercises to prevent pulmonary atelectasis in lung cancer patients. Participants who underwent anatomic pulmonary resection and received postoperative rehabilitation interventions that included breathing exercises as a component were included in this review. The study quality and risks of bias were measured with the GRADE and Cochrane Collaboration tools, and statistical analysis was performed utilizing RevMan 5.3 software.

**Results:**

The incidence of atelectasis was significantly lower in the postoperative rehabilitation intervention group (OR = 0.35; 95% CI, 0.18 to 0.67; I2 = 0%; *P* = 0.67) than in the control group. The patients who underwent the postoperative rehabilitation program that included breathing exercises (intervention group) had higher forced vital capacity (FVC) scores (MD = 0.24; 95% CI, 0.07 to 0.41; I^2^ = 73%; *P* = 0.02), forced expiratory volume in one second (FEV1) scores (MD = 0.31; 95% CI, 0.03 to 0.60; I^2^ = 98%; *P* < 0.01) and FEV1/FVC ratios (MD = 9.09; 95% CI, 1.50 to 16.67; I^2^ = 94%; *P* < 0.01).

**Conclusion:**

Postoperative rehabilitation interventions that included breathing exercises decreased the incidence rate of atelectasis and improved lung function by increasing the FVC, FEV1, and FEV1/FVC ratio.

**Supplementary Information:**

The online version contains supplementary material available at 10.1186/s12890-023-02563-9.

## Introduction

According to the World Health Organization, cancer is a leading cause of death worldwide, claiming nearly 10 million lives in 2020 [1]. In both sexes, the most common cancer was lung cancer, accounting for 11.6% of total cancer cases, and it was fatal with a mortality rate of 18.4% of total cancer deaths in 2018 [2]. Surgery is an important treatment option in which doctors excise the cancer tissue from patients [3]. However, postoperative pulmonary complications (PPCs) remain difficult clinical issues hindering recovery. The incidence of PPCs is approximately 32–39% depending on individual biases, such as in health conditions and surgical methods [4, 5]. Pneumonia and atelectasis are the most common PPCs after lung resection surgery [6]. Even mild PPCs can lead to serious clinical problems including increased early postoperative mortality and a prolonged length of stay in the intensive care unit or hospital [7]. Therefore, it is of great clinical value to determine specific rehabilitation interventions to decrease the incidence of PPCs and improve lung function in lung cancer patients after anatomic pulmonary resection.

Some preoperative pulmonary rehabilitation programs have been proven to play an important role in functional recovery. For example, exercise-based programs that included breathing exercises could improve exercise tolerance, and muscle strength, and enhance postoperative recovery [8]. Other postoperative training such as inspiratory muscle training and exercise training was associated with less sedentary activity and prevented a decline in physical activity [9]. Breathing exercises were also demonstrated to improve lung function and quality of life [10]. However, the evidence was controversial in some finer details. Although clinicians used breathing exercises as part of the treatment regimen for lung cancer patients after surgery, some studies found that preoperative interventions could not reduce the incidence of PPCs, such as pneumonia and atelectasis [11, 12].

Previous meta-analyses have quantified and drawn conclusions about the effects of preoperative breathing exercises on PPCs [13–16], while others were focused on perioperative pulmonary rehabilitation interventions [11, 12, 17, 18]. However, there has been no meta-analysis specifically focusing on postoperative interventions in addition to a regular rehabilitation program in reducing the incidence of atelectasis in lung cancer resection patients. Therefore, the purpose of this systematic review was to analyze the postoperative rehabilitation programs that include breathing exercises and determine whether they were effective in reducing the incidence of atelectasis and improving lung function.

## Methods

This systematic review was reported based on guidelines of the Preferred Reporting Items for Systematic Reviews and Meta-Analysis Protocol Statement (PRISMAP) [19] and the procedures used in this systematic review were based on the Cochrane Handbook for Systematic Reviews of Interventions [20]. The scope of the systematic review was specified using the PICOS (participants, interventions, comparisons, outcomes, study type) framework. The PICOS question was: in patients with pulmonary cancer who underwent surgical resection, do postoperative rehabilitation programs that include breathing exercises decrease the incidence rate of pulmonary atelectasis, compared to the regular rehabilitation program?

In this review, we defined the control group as patients who underwent regular rehabilitation programs, including medication management, physiotherapy, and health education. The intervention group was defined as patients who underwent postoperative rehabilitation programs that included any breathing exercises as a component, such as inspiratory muscle training, abdominal breathing training, and the utility of assistive training devices related to breathing.

All included studies were randomized controlled trials. We developed a review protocol using the planned analysis approach. This systematic review was registered in PROSPERO, and the registration number was CRD42022343946.

### Inclusion criteria

Studies were eligible if participants with lung cancer underwent any type of surgical resection (all types of surgery were included); a comparison between postoperative rehabilitation programs including breathing exercises at any intensity and regular rehabilitation program was conducted; the incidence of pulmonary atelectasis as outcome measurement was provided; and the methods study type was a randomized controlled trial. Additionally, the included studies must have been published in peer-reviewed journals with full texts available either in English or Chinese.

### Exclusion criteria

Studies were excluded if they were case reports, case series, or observational studies; if the reason for surgery was not lung cancer; if the participants from the intervention group did not receive any postoperative breathing training; if the language of the studies was neither English nor Chinese; or if they did not include the primary outcomes set by the protocol.

### Outcomes

The primary outcome was the incidence rate of atelectasis after surgery. The secondary outcomes were FEV1, FVC, and the FEV1/FVC ratio, which are important indicators for factors to the prognosis of atrophic pulmonary resection [5, 21, 22].

### Search strategy

One of the authors (JW) systematically and comprehensively searched the Cochrane Library, PubMed, EMBASE, and Web of Science in English, and CNKI and Wanfang in Chinese for studies published during the last decade (from 2012 to 2022) using medical and random terms. The authors also searched ClinicalTrials.gov and the World Health Organization International Clinical Trials Registry Platform to identify ongoing or unpublished eligible trials.

### Study selection

**Fig. 1:**
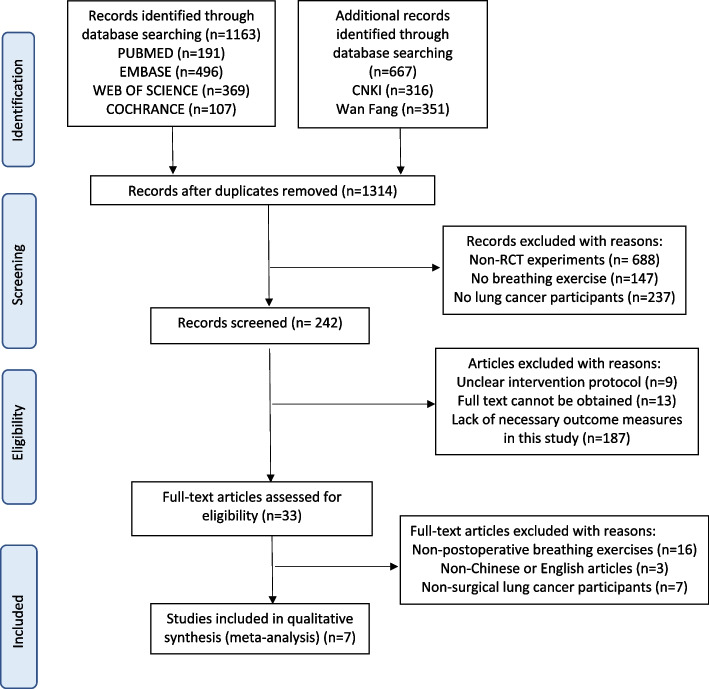
Flow of the search strategy

The search for articles was performed separately by two authors (JW, ND), and the results were imported into EndNote. After the removal of duplicates among the retrieved articles, the title and abstract of the articles were independently reviewed by two authors (JW, HH) to complete the rough screening process. If the results were inconsistent, the third author (FQ) resolved the issue through consensus. Finally, after reading the full text of the retrieved RCT articles, another researcher was consulted to make inclusion decisions if needed. All the processes are shown in a flow chart (Fig. 1).

### Data collection process

All the data extraction processes were performed by two authors separately (JW, ND); the third researcher (FQ) was consulted if there were disagreements. If a study mentioned an outcome of interest without providing details, we contacted the author for the data. Disagreements were resolved by consensus.

### Risk of bias assessment in individual studies

The quality of individual studies was assessed by two authors separately (ND, QL), using the Cochrane Collaboration risk of bias tool, and the quality of evidence for outcomes was examined using the Grading of Recommendations Assessment, Development, And Evaluation (GRADE) approach [23].

### Data synthesis

Statistical analysis was performed by utilizing RevMan 5.3.3 software. This systematic review used odds ratios and their associated 95% confidence intervals to assess the primary outcome of, postoperative complications. The mean difference value was used to assess the secondary outcomes. If I^2^ > 50 and the *p*-value was less than 0.05, we considered the result to be heterogeneous and applied a random-effects model [24]. Accordingly, a subgroup was applied to analyze the heterogeneity. If I^2^ < 50 and the *p*-value was greater than 0.05, no significant heterogeneity was detected, and we applied a fixed-effects model.

### Subgroup analysis

To determine intervention effects for different types of atelectasis, we intended to perform a subgroup analysis. However, the retrieved studies did not provide us with enough information about the types of atelectasis observed. Thus, the subgroup analysis was discontinued.

### Sensitivity analysis

In this systematic review, we intended to conduct sensitivity analyses by excluding articles with a high risk of bias, which often has some different characteristics. In this way, we could use the random-effects model rather than the fixed-effects model. Alternatively, each study was excluded one by one, and the remaining studies were pooled to determine whether the results varied significantly.

## Results

### Flow of trials through the review process

The search strategy identified 1,830 records, of which 516 were found to be duplicates. After screening titles, abstracts, and reference lists, 33 potentially relevant full articles were screened. After evaluating full-text articles, 26 studies failing to meet the inclusion criteria were excluded, and 7 studies were included in this systematic review. Figure 1 outlines the flow of studies through the review process.

### Characteristics of the included trials

The 7 included trials involved 569 participants and investigated the effect of postoperative rehabilitation programs on the incidence of atelectasis after lung resection. FEV1 was included in 5 studies [25–29], FVC in 3 studies [26, 27, 29] and FEV1/FVC in 2 studies [28, 29]. Six studies [25–27, 29–31] performed postoperative rehabilitation programs with at least one breathing exercise device. Two studies performed inspiratory muscle training [25, 30]. The initial resistance load was set at 30% and the load was gradually increased until the patient was discharged. The load of the training method was adjusted according to the patient’s tolerance. Additional relevant characteristics of the included studies are shown in Table 1. GRADE evidence quality ratings are shown in Table 2.

**Table 1:** Characteristics of included trials

			Intervention group			Control group			
study	country	Sample size	types	Intensity	frequency	types	intensity	frequency	Outcome
Zou et al., 2022 [29]	China	90	ABCDEF program : Acapella positive vibration pressure training, breathing exercises, cycling training, dance in the square, education, and follow-up	Gradually increase the intensity, the lowest level at the beginning	Each for 15–20 min or 10–15 min, Twice or thrice a day (beginning at the perioperative period and lasting for 3 months)	Breathe deeply, surgical cooperation, oral care, turning over, oxygen inhalation, and atomization inhalation, shrinking lip breathing, blowing balloons, effective cough training, and climbing stairs	NR	NR	FEV1, FVC, FEV1/FVC ratio, 6 MWT, Borg score, the incidence of postoperative complications, length of indwelling chest tube, and length of postoperative stay
Brocki et al., 2016 [30]	Denmark	70	IMT	30% of MIP	15 reps/set, 2 sets/session, twice daily (1 working day before surgery and continuing for 2 weeks after surgery)	standard physiotherapy Treatment: deep inspiration, coughing, huffing technique	NR	NR	MIP, MEP,6MWT, Dyspnea after–before 6MWT, Oxygen saturation after 6MWT, FVC% predicted, FEV1% predicted, FEV1/FVC ratio
Liu et al., 2021 [25]	China	54	IMT and aerobic exercise	The initial resistance load was set at 30% MIP	IMT: 30 reps/set, 2 sets/day aerobic exercise: 60-min/day (beginning on the day of chest tube removal to 6 weeks after discharge)	standard pulmonary rehabilitation program: smoking cessation and breathing control, upper and lower limb exercises, incentive spirometry, lung expansion therapy, and airway clearance therapy	NR	NR	MIP, MEP,6MWT, Lung expansion Volume
Zhou et al., 2022 [28]	China	86	relaxing and exercising the intercostal muscles, thoracic costal joint, and abdominal breathing muscles education, aerobic exercise, and breathing exercises	VAS was 2–6 points	NR (after surgery and last for 28 days)	Routine program: Education, aerobic exercise and breathing exercises	NR	Breathing exercise: 9–15 min/set, 2 times/day aerobic exercise: 15 min/set, 2 sets/day	PEF, FEV1, FEV1/FVC, FVC,6MWT, Borg scale score
Li et al., 2018 [31]	China	69	standard treatment and positive vibration pressure training	starting from the minimum resistance on the positive vibration pressure training	10–20 reps/set, 1 set every 2 h (beginning at the perioperative period and lasting for 1 month)	standard clinic care; Preoperative education, abdominal breathing training, cough training, pain management	NR	NR	PPCs, the duration of total hospital stay and postoperative hospital stay, The drug cost, FEV1, PEF
Shen et al., 2021 [26]	China	92	Convention breathing exercises and breathing exercise device	NR	10–15 reps/ set, 6 sets/ day (beginning after surgery and lasting for 3 months)	Convention breathing exercises: deep coughing, pursed lip breathing, abdominal breathing, balloon blowing	NR	NR	FEV1, FVC, MVV, Borg score, 6MWT, PPCs
Yang et al., 2021 [27]	China	110	Respirator exercises: pursed lip breathing, balloon blowing	NR	10–15 reps/set, 2 sets/day (beginning after surgery and lasting for 3 months)	routine clinic care: Monitor vital signs, guide medication, respiratory management, diet management, pain management, and psychological care	NR	NR	FEV1, FVC, FEV1/FVC oxygen saturation, blood partial pressure of oxygen, quality of life, PPCs

*Abbreviations: NR* Do not know, *FEV1* the forced expiratory volume in the first second, *FVC* Forced vital capacity, *6MWT* 6 min walking distance, *PPCs* Postoperative pulmonary complications, *NRS* Numeric Rating Scale, *IMT* Inspiratory muscle training, *MIP* Maximal expiratory pressure, *MEP* Maximal inspiratory pressure, *VAS* Visual analog scale, *QoL* Quality of life

**Table 2:**
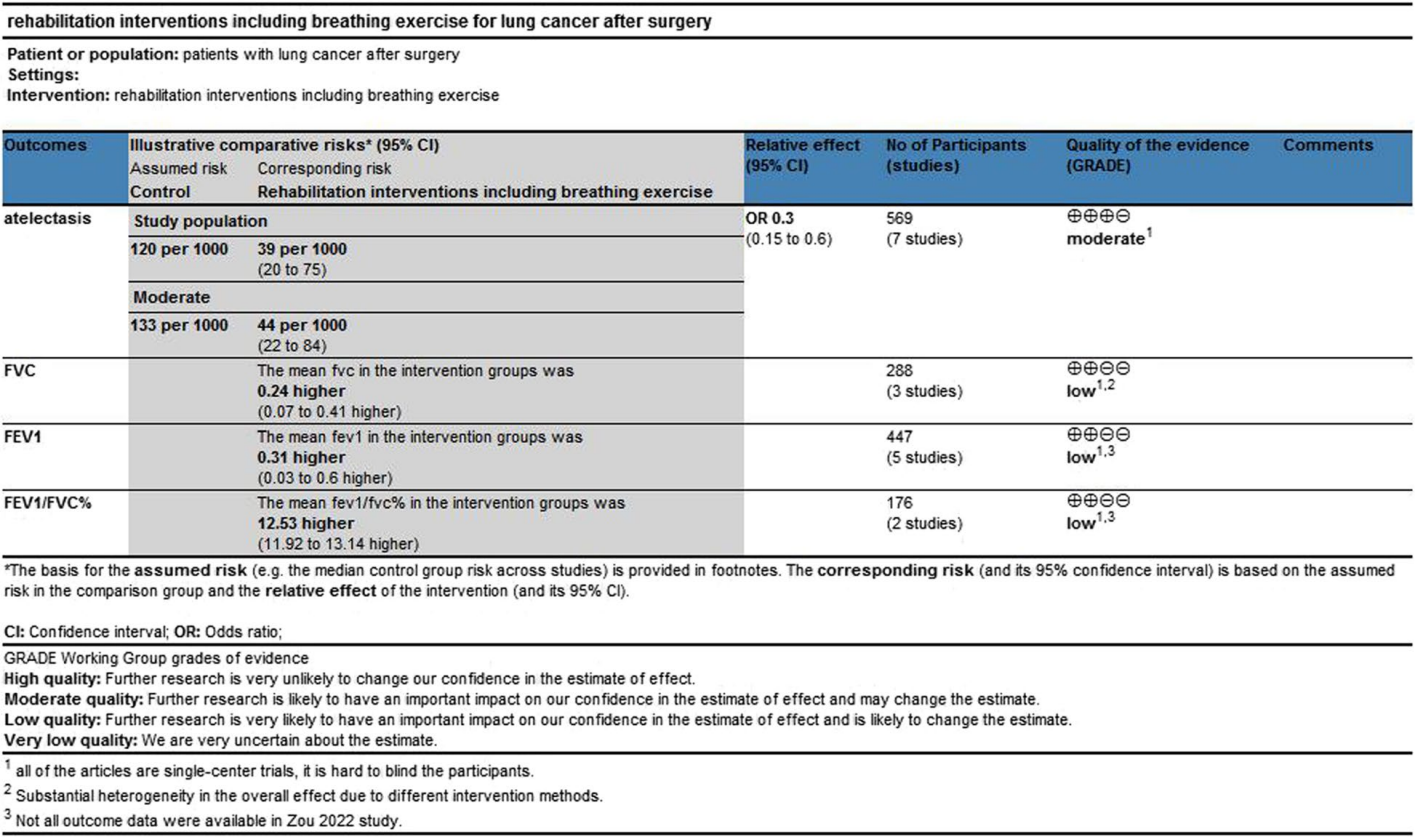
GRADE evidence quality ratings

### Risk of bias assessment

**Fig. 2:**
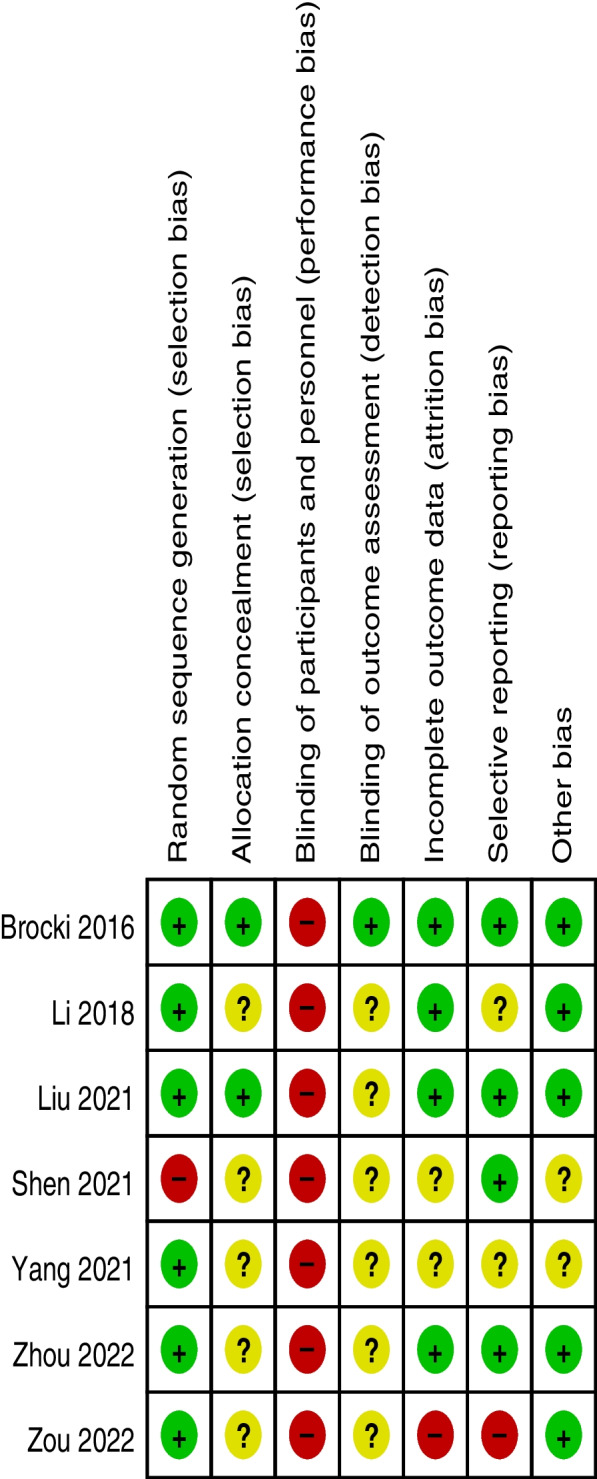
Risk of bias summary respectively

**Fig. 3:**
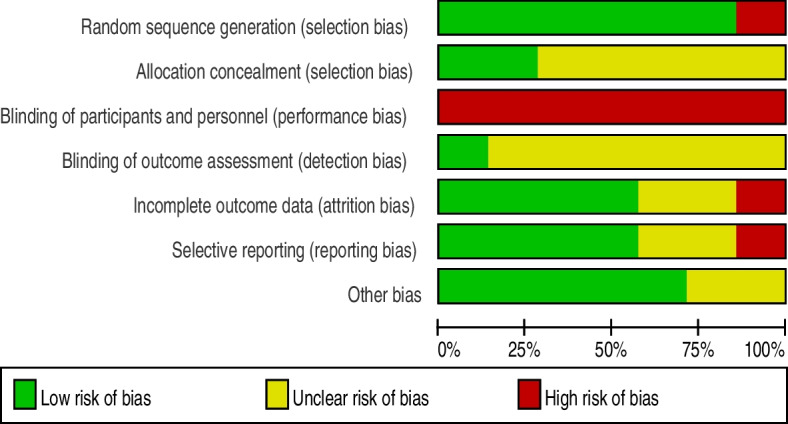
Risk of bias summary generally

The outcomes of the quality assessment of the studies were conducted in Review Manager 5.3 software according to the quality assessment judgment criteria. The risk of bias was assessed as low, high, or unclear risk. None of the studies described detailed information about blinding participants; all the articles were single-center studies, and it was hard to blind the participants, so all studies had a high risk of performance bias. Six trials [25–28, 30, 31] had complete outcomes data, except for Zou et al.’s study [29]. Six trials [25, 27–31] were RCTs with a clear method of randomization, except Shen et al.’s study [26]. Brocki et al.’s study [30] was the only study that clearly explained assessors blinding, thus we only considered it as low risk of detection bias. Additional detailed information about the risk of bias assessments is shown in Figs. 2, 3.

### Primary outcome

#### Incidence of atelectasis

**Fig. 4:**
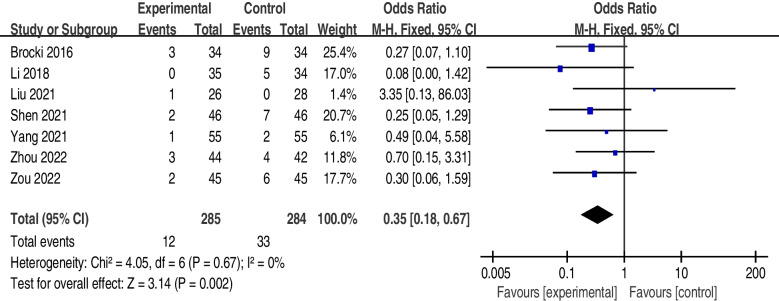
The forest plot showing OR (95% CI) of atelectasis incidence after implementation of postoperative rehabilitation interventions that include breathing exercise

The effect of postoperative rehabilitation programs on atelectasis was examined by pooling the data from all 7 trials [25–31]. The result shows that there is no heterogeneity, so a fixed-effects model was applied. The incidence of atelectasis was significantly lower after postoperative rehabilitation programs (OR = 0.35; 95% CI, 0.18 to 0.67; I^2^ = 0%; *P* = 0.67) compared with the control group (Fig. 4).

### Secondary outcomes

#### FVC

**Fig. 5:**
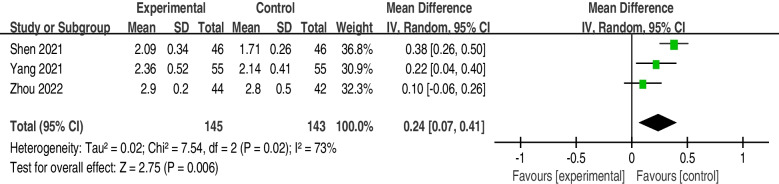
FVC forest plot showing the mean difference (95% CI) of the effect of postoperative rehabilitation interventions that include breathing exercise on FVC

The effect of breathing exercises on FVC was examined by pooling data from 5 trials [26–30]. However, complete data extraction failed in the studies of Zou et al. [29] and Brocki et al [30]. Therefore, a total of 3 trials [26–28] were included in the meta-analysis. When a random-effects model was applied, postoperative rehabilitation programs can improve the score of FVC (MD = 0.24; 95% CI, 0.07 to 0.41; I^2^ = 73%; *P* = 0.02) (Fig. 5).

#### FEV1

**Fig. 6:**
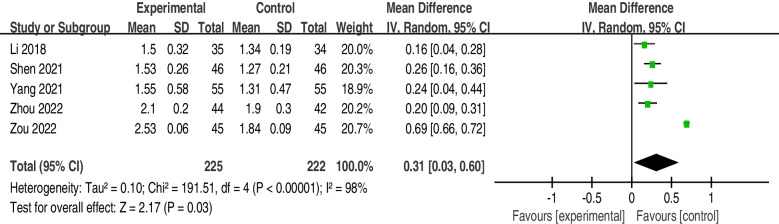
FEV1 forest plot showing the mean difference (95% CI) of the effect of postoperative rehabilitation interventions that include breathing exercise on FEV1

6 trials collected the data on FEV1, but we failed to extract the data on FEV1 from Brocki et al. [30] and pooled the data from the other 5 trials [26–29, 31]. There was a difference between the experimental group and intervention group (MD = 0.31; 95% CI, 0.03 to 0.60; I^2^ = 98%; *P* < 0.01) (Fig. 6).

#### FEV1/FVC ratio

**Fig. 7:**
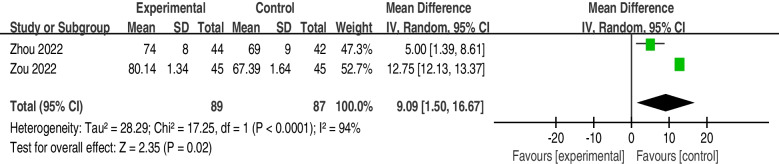
FEV1/FVC forest plot showing the mean difference (95%) of the effect of postoperative rehabilitation interventions that include breathing exercise on the FEV1/FVC ratio

Four articles [27–30] reported the value of the FEV1/FVC ratio. We excluded two articles from Brocki et al. [30] and Yang et al., [27] because of a lack of original data. Thus, the effect of postoperative rehabilitation programs on the FEV1/FVC ratio was examined by pooling data from 2 trials [28, 29]. The result shows that postoperative rehabilitation programs can increase the grade of FEV1/FVC Ratio (MD = 9.09; 95% CI, 1.50 to 16.67; I^2^ = 94%; *P* < 0.01) (Fig. 7).

## Discussion

This systematic review included 7 studies in total that reported the incidence of atelectasis after postoperative intervention as one of the outcome measurements. By analyzing these RCT articles, we found that lung cancer patients undergoing surgical resection would benefit from postoperative rehabilitation programs that include breathing exercises to decrease their incidence of atelectasis, a common PPC. In addition, the heterogeneity was negligible for this indicator. We also performed a sensitivity analysis using the leave-one-out method and excluding one study at a time to change the fixed model to a random-effects model. The results remained robust (Sup. 1).

A previous meta-analysis also assessed the effects of breathing exercises on PPCs in lung cancer patients. Wang et al. collected data through 20 December 2017 and grouped the breathing exercises based on different stages including preoperative, postoperative, and perioperative in their retrieved studies. [12] However, their data analysis was debatable, with a view to the inclusion criteria of postoperative rehabilitation interventions and the computation methods of incidence of atelectasis. In our meta-analysis, we reviewed the research during the last decade through 2022 and focused on postoperative rehabilitation interventions that included breathing exercises as a component. Additionally, we used the incidence of atelectasis as the primary outcome. Therefore, our review was more focused on postoperative interventions and their preventive effects on atelectasis. Another reason for selecting postoperative rehabilitation was the unique medical-social phenomenon in China. In Lai et al.’s study, 22 eligible patients refused to participate in preoperative rehabilitation treatment. [32] The primary causes for lung patients’ reluctance were the lack of public health consciousness and financial problems caused by a prolonged length of stay in the hospital, which is a common social issue in China and other developing countries [33]. Lung cancer patients hope to undergo the operation as soon as possible when they are admitted to the hospital rather than receive preoperative rehabilitation. Therefore, we considered it more practical to study postoperative rehabilitation in developing countries. In the future, as health care and awareness improve, our research will focus more on not only postoperative but also perioperative breathing exercises, which are also of great clinical significance [8, 31].

According to our results, we had positive findings for FEV1, the FEV1/FVC ratio, and FVC, which indicated that a postoperative rehabilitation program including breathing exercises would help lung cancer patients improve their lung function. However, the heterogeneity among those values was significant. To analyze the heterogeneity source of FEV1, we conducted a subgroup analysis for possible influencing factors, including intervention types, intervention timing, and others. We found that the use of Acapella could be an influencing factor (Sup. 2). The intervention group participants in the studies of Li et al. and Zhou et al. were provided with Acapella, a widely used breathing training device in pulmonary rehabilitation. [28, 31] Only two from Zhou et al. and Zou et al. included reported the FEV1/FVC ratio. [28, 29] Thus, we were unable to conduct a subgroup analysis. We reviewed the details of the articles and discovered that the risk of bias could be the primary cause of the heterogeneity. We evaluated the risks of attribution bias and reporting bias in the study by Zou et al., which were both high. [29] This study mentioned FVC in the abstract as an outcome measure, but no data were found in the results section, which was considered incomplete outcome data. On the other hand, the risks of bias in the above two aspects in the study by Zhou et al. [28] were low. The substantial difference in the risks of bias could also be the reason why Zou et al. [29] found much greater positive effects between the study groups than the study by Zhou et al. [28] In terms of FVC, the heterogeneity could derive from the interventions provided to the control group. The control group participants in the study by Yang et al. [27] did not receive any physiotherapy, while the studies by Shen et al. [26] and Zhou et al. [28] provided a common physiotherapy intervention to the participants in the control group.

Finally, for this meta-analysis, explicit eligibility criteria were established, and a meticulous search of the different databases was performed. We assessed the risk of bias to determine the reliability of the evidence. Additionally, the included studies were limited to the highest standard of evidence, only RCTs. However, the secondary outcome findings should be generalized carefully due to the limited number of cases, the deficiency in the blinding of therapists and patients, and the diversity in the content of breathing exercise programs in the retrieved studies. To conclude, this meta-analysis revealed that postoperative rehabilitation interventions that include breathing exercises could decrease the incidence of atelectasis in patients with lung cancer after surgery. Additionally, it provided a clinical basis for future considerations on whether postoperative rehabilitation interventions that include breathing exercises should be implemented after surgery.

## Study limitations

The main limitation of this review was the obvious heterogeneity in the secondary outcomes of FVC, FEV1 and the FVE1/FVC ratio. However, there were only a few studies that reported FVC and the FEV1/FVC ratio, and it was difficult to conduct subgroup analysis. Thus, we compared the detailed information, including research design, intervention methods, and data collection. Second, no studies performed well in preventing performance bias, and only the study by Brocki et al. performed well in blinding for outcome assessments. [30] This led to a low level of evidence. Third, the rehabilitation intervention in the study by Brocki et al. was started one day before surgery; however, since the majority part of the intervention was performed after the surgery, [30] we still considered it to be a postoperative intervention.

Additionally, it was difficult to determine the effect of breathing exercises alone without involving any other general rehabilitation interventions given the objective facts in the clinical settings and ethical considerations. We cannot deduce whether these breathing exercises will work independently from any other rehabilitation interventions.

## Supplementary Information

**Additional file 1:** **Sup. 1. **The forest plot showing OR (95% CI) of atelectasis incidence after implementation postoperative rehabilitation programs (random model).

**Additional file 2:** **Sup. 2.** FEV1 forest plot showing the total mean difference (95% CI) of the effect of postoperative rehabilitation interventions that include breathing exercise on FEV1 subgroup score (subgroup: with or without the device of Acapella).

## Data Availability

Detailed information on search strategies and other relevant material that support the findings of this systematic review is available from the corresponding author upon reasonable request.

## Abbreviations

CI: Confidence interval
RCTs: Randomized controlled trials
PPCs: Postoperative pulmonary complications
6MWT: The 6-min walk test
FVC: Forced vital capacity
FEV1: Forced expiratory volume in 1-s
