# Supplementary material for: The effectiveness of postoperative rehabilitation interventions that include breathing exercises to prevent pulmonary atelectasis in lung cancer resection patients: a systematic review and meta-analysis
Source: BMC Pulm Med. 2023 Jul 27;23:276. doi: 10.1186/s12890-023-02563-9 (PMC10375623; doi:10.1186/s12890-023-02563-9)
Supplement: Supplementary file 1 — Additional file 1: Sup. 1. The forest plot showing OR (95% CI) of atelectasis incidence after implementation postoperative rehabilitation programs (random model). [file 12890_2023_2563_MOESM1_ESM.docx]

**Sup. 1** The forest plot showing OR (95% CI) of atelectasis incidence after implementation postoperative rehabilitation programs (random model).

|  | **Experimental** | | **Control** | | | **Odds Ratio** | **Odds Ratio** |
| --- | --- | --- | --- | --- | --- | --- | --- |
| **Study or Subgroup** | **Events** | **Total** | **Events** | **Total** | **Weight** | **M-H, Random,95% Cl**  0.05  0.1  1  10  200 | **M-H, Random, 95% CI** |
| Brocki 2015 | 3 | 34 | 9 | 34 | 24.8% | 0.27 [0.07 , 1.10] |  |
| Li 2018 | 0 | 35 | 5 | 34 | 5.7% | 0.08 [0.00 , 1.42] |  |
| Liu 2021 | 1 | 26 | 0 | 28 | 4.7% | 3.35 [0.13 , 86.03] |  |
| Shen 2021 | 2 | 46 | 7 | 46 | 18.5% | 0.25 [0.05 , 1.29] |  |
| Yang 2021 | 1 | 55 | 2 | 55 | 8.3% | 0.49 [0.04 , 5.58] |  |
| Zhou 2021 | 3 | 44 | 4 | 42 | 20.2% | 0.70 [0.15 , 3.31] |  |
| Zou 2021 | 2 | 45 | 6 | 45 | 17.9% | 0.30 [0.06 , 1.59] |  |
|  |  |  |  |  |  |  |  |
| **Total(95%Cl)** |  | **285** |  | **284** | **100%** | **0.36 [0.18 , 0.73]** |  |
| Total events | 12 |  | 33 |  |  |  |  |
| Heterogeneity: Tau^2^ = 0.00; Chi^2^ =4.05 , df = 6 (P = 0.67); I^2^ = 0%  Test for overall effect: Z = 2.84 (P = 0.004) | | | | | | | Favours [experimental] Favours [control] |
