# Supplementary material for: The effectiveness of postoperative rehabilitation interventions that include breathing exercises to prevent pulmonary atelectasis in lung cancer resection patients: a systematic review and meta-analysis
Source: BMC Pulm Med. 2023 Jul 27;23:276. doi: 10.1186/s12890-023-02563-9 (PMC10375623; doi:10.1186/s12890-023-02563-9)
Supplement: Supplementary file 2 — Additional file 2: Sup. 2. FEV1 forest plot showing the total mean difference (95% CI) of the effect of postoperative rehabilitation interventions that include breathing exercise on FEV1 subgroup score (subgroup: with or without the device of Acapella). [file 12890_2023_2563_MOESM2_ESM.docx]

|  | **Experimental** | | | **Control** | | | | **Mean Difference** | **Mean Difference** |
| --- | --- | --- | --- | --- | --- | --- | --- | --- | --- |
| **Study or Subgroup** | **Mean** | **SD** | **Total** | **Mean** | **SD** | **Total** | **Weight** | **IV, Fixed, 95% CI** | **V, Fixed, 95%** |
| **2.6.1 device** |  |  |  |  |  |  |  |  | 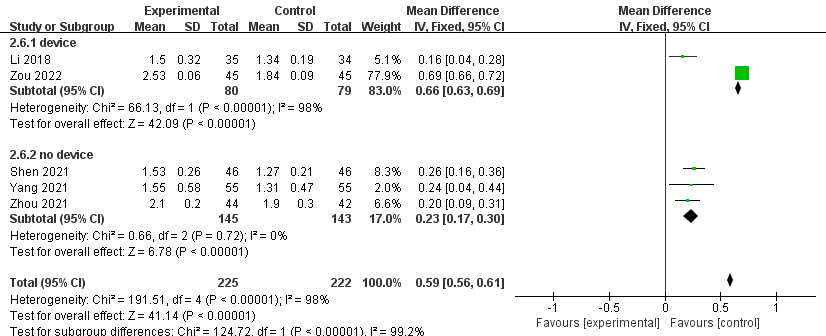  -1  -0.5  0  0.5  1 |
| Li 2018 | 1.5 | 0.32 | 35 | 1.34 | 0.19 | 34 | 5.1% | 0.16 [0.04, 0.28] |  |
| Zou 2021 | 2.53 | 0.06 | 45 | 1.84 | 0.09 | 45 | 77.9% | 0.69 [0.66, 0.72] |  |
| **Subtotal (95% CI)** |  |  | **80** |  |  | **79** | **83.0%** | **0.66 [0.63, 0.69]** |  |
| Heterogeneity: Chi^2^ = 66.13, df= 1 (P < 0.00001); I^2^ = 98%  Test for overall effect: Z = 42.09 (P < 0.00001) | | | | | | | | |  |
|  | | | | | | | | |  |
| **2,6,2 no device** | | | | | | | | |  |
| Shen 2021 | 1.53 | 0.26 | 46 | 1.27 | 0.21 | 46 | 8.3% | 0.26 [0.04, 0.36] |  |
| Yang 2021 | 1.55 | 0.58 | 55 | 1.31 | 0.47 | 55 | 2.0% | 0.24 [0.04, 0.44] |  |
| Zhou 2021 | 2.1 | 0.2 | 44 | 1.9 | 0.3 | 42 | 6.6% | 0.20 [0.09, 0.31] |  |
| **Subtotal (95% CI)** |  |  | **145** |  |  | **143** | **17.0%** | **0.23 [0.17, 0.30]** |  |
| Heterogeneity: Chi^2^= 0.66, df= 2 (P = 0.72): I^2^ = 0%  Test for overall effect: Z = 6.78 (P < 0.00001) | | | | | | | | |  |
|  |  |  |  |  |  |  |  |  |  |
| **Total(95%Cl)** |  |  | **225** |  |  | **222** | **100.0%** | **0.59 [0.17, 0.30]** |  |
| Heterogeneity: Chi^2^ = 191.51, df= 4 (P < 0.00001); I^2^ = 98%  Test for overall effect: Z = 41.14 (P < 0.00001)  Test for subgroup differences: Chi^2^ = 124.72. df= 1 (P <0.00001). I^2^ = 99.2% | | | | | | | | | Favours [experimental] Favours [control] |

**Sup. 2** FEV1 forest plot showing the total mean difference (95% CI) of the effect of postoperative rehabilitation interventions that include breathing exercise on FEV1 subgroup score (subgroup: with or without the device of Acapella)
